# dl-Asparaginium nitrate

**DOI:** 10.1107/S1600536809031730

**Published:** 2009-08-19

**Authors:** Nabila Moussa Slimane, Aouatef Cherouana, Lamia Bendjeddou, Slimane Dahaoui, Claude Lecomte

**Affiliations:** a Laboratoire de Chimie Moléculaire, du Contrôle, de l’Environnement et des Mesures Physico-Chimiques, Faculté des Sciences, Département de Chimie, Université Mentouri de Constantine, 25000 Constantine, Algeria; b Cristallographie, Résonance Magnétique et Modélisation (CRM2), Université Henri Poincaré, Nancy 1, Faculté des Sciences, BP 70239, 54506 Vandoeuvre lès Nancy CEDEX, France

## Abstract

In the title compound, C_4_H_9_N_2_O_3_^+^·NO_3_^−^, alternatively called (1*RS*)-2-carbamoyl-1-carboxy­ethanaminium nitrate, the asymmetric unit comprises one asparaginium cation and one nitrate anion. The strongest cation–cation O—H⋯O hydrogen bond in the structure, together with other strong cation–cation N—H⋯O hydrogen bonds, generates a succession of infinite chains of *R*_2_^2^(8) rings along the *b* axis. Additional cation–cation C—H⋯O hydrogen bonds link these chains into two-dimensional layers formed by alternating *R*_4_^4^(24) and *R*_4_^2^(12) rings. Connections between these layers are provided by the strong cation–anion N—H⋯O hydrogen bonds, as well as by one weak C—H⋯O inter­action, thus forming a three-dimensional network. Some of the cation–anion N—H⋯O hydrogen bonds are bifurcated of the type *D*—H⋯(*A*_1_,*A*_2_).

## Related literature

dl-Asparagine has been used in growth media for bacteria, see: Gerhardt & Wilson (1948 ▶); Palleroni *et al.* (1973 ▶); Wagtendonk *et al.* (1963 ▶). For related structures, see Aarthy *et al.* (2005 ▶); Anitha *et al.* (2005 ▶); Arnold *et al.* (2000 ▶); Flaig *et al.* (2002 ▶); Kartha & de Vries (1961 ▶); Ramanadham *et al.* (1972 ▶); Smirnova *et al.* (1990 ▶); Verbist *et al.* (1972 ▶); Wang *et al.* (1985 ▶); Weisinger-Lewin *et al.* (1989 ▶); Yamada *et al.* (2007 ▶). For hydrogen bonding, see: Desiraju & Steiner (1999 ▶). For hydrogen-bond morifs, see: Bernstein *et al.* (1995 ▶); Etter *et al.* (1990 ▶).
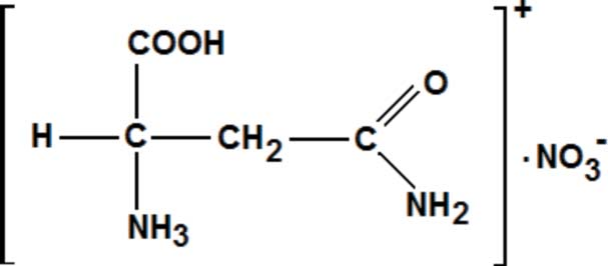


## Experimental

### 

#### Crystal data

C_4_H_9_N_2_O_3_^+^·NO_3_^−^
*M* *_r_* = 195.14
Monoclinic, 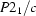

*a* = 7.923 (2) Å
*b* = 9.608 (2) Å
*c* = 10.613 (3) Å
β = 107.105 (2)°
*V* = 772.2 (3) Å^3^
*Z* = 4
Mo *K*α radiation
μ = 0.16 mm^−1^
*T* = 100 K
0.30 × 0.20 × 0.09 mm

#### Data collection

Oxford Diffraction Xcalibur–Sapphire2 CCD diffractometer
Absorption correction: gaussian (*CrysAlis RED*; Oxford Diffraction, 2008 ▶) *T*_min_ = 0.966, *T*_max_ = 0.991
19446 measured reflections
2236 independent reflections
1804 reflections with *I* > 2σ(*I*)
*R*_int_ = 0.036

#### Refinement

*R*[*F* ^2^ > 2σ(*F* ^2^)] = 0.035
*wR*(*F* ^2^) = 0.087
*S* = 1.07
2236 reflections
136 parameters
H atoms treated by a mixture of independent and constrained refinement
Δρ_max_ = 0.45 e Å^−3^
Δρ_min_ = −0.19 e Å^−3^

### 

Data collection: *CrysAlis CCD* (Oxford Diffraction, 2008 ▶); cell refinement: *CrysAlis RED* (Oxford Diffraction, 2008 ▶); data reduction: *CrysAlis RED*; program(s) used to solve structure: *SIR92* (Altomare *et al.*, 1993 ▶); program(s) used to refine structure: *SHELXL97* (Sheldrick, 2008 ▶); molecular graphics: *ORTEPIII* (Farrugia, 1997 ▶) and *Mercury* (Macrae *et al.*, 2006 ▶); software used to prepare material for publication: *PLATON* (Spek, 2009 ▶).

## Supplementary Material

Crystal structure: contains datablocks global, I. DOI: 10.1107/S1600536809031730/fb2157sup1.cif

Structure factors: contains datablocks I. DOI: 10.1107/S1600536809031730/fb2157Isup2.hkl

Additional supplementary materials:  crystallographic information; 3D view; checkCIF report

## Figures and Tables

**Table 1:** Hydrogen-bond geometry (Å, °)

*D*—H⋯*A*	*D*—H	H⋯*A*	*D*⋯*A*	*D*—H⋯*A*
O1—H1⋯O3^i^	0.845 (16)	1.736 (16)	2.571 (2)	169.0 (17)
N2—H1*N*⋯O4^ii^	0.899 (15)	1.962 (15)	2.822 (2)	159.6 (13)
N2—H2*N*⋯O3	0.907 (15)	2.406 (16)	2.965 (2)	119.9 (12)
N2—H2*N*⋯O5	0.907 (15)	2.233 (14)	3.024 (2)	145.4 (13)
N2—H2*N*⋯O6	0.907 (15)	2.474 (15)	3.039 (2)	120.7 (11)
N2—H3*N*⋯O4^iii^	0.903 (14)	2.454 (14)	3.157 (2)	135.0 (12)
N2—H3*N*⋯O6^iii^	0.903 (14)	2.068 (15)	2.957 (2)	168.3 (14)
N3—H5*N*⋯O2^iv^	0.893 (16)	2.064 (15)	2.924 (2)	161.4 (15)
C3—H3⋯O5^v^	0.99	2.36	3.086 (2)	130
C3—H4⋯O2^v^	0.99	2.39	3.313 (2)	156

Symmetry codes: (i) 
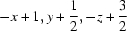
; (ii) 
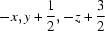
; (iii) 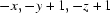; (iv) 
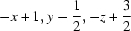
; (v) 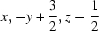.

**Fig. 1.**
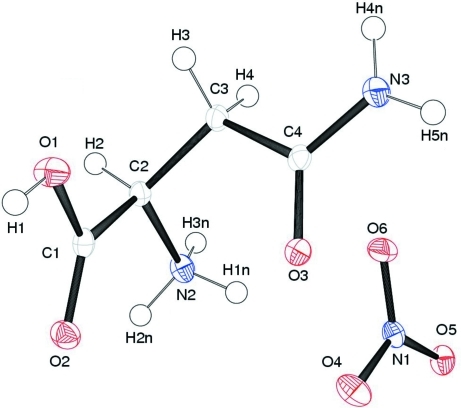
The title molecule with the atom-numbering scheme. The displacement ellipsoids are drawn at the 50% probability level

**Fig. 2.**
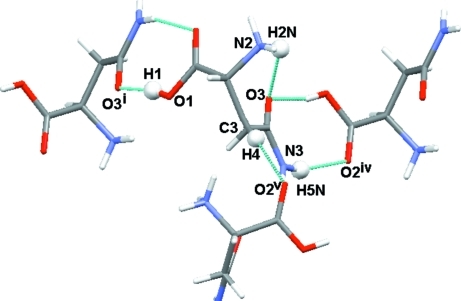
The cation-cation hydrogen bonds. Symmetry codes: (i) -x+1, y+1/2, -z+3/2; (iv) -x+1, y-1/2, -z+3/2; (v) x, -y+3/2, z-1/2

**Fig. 3.**
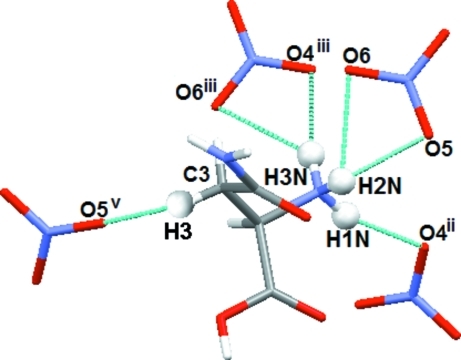
The cation-anion hydrogen bonds. Symmetry codes: (ii) -x, y+1/2, -z+3/2; (iii) -x, -y+1, -z+1; (v) x, -y+3/2, z-1/2

**Fig. 4.**
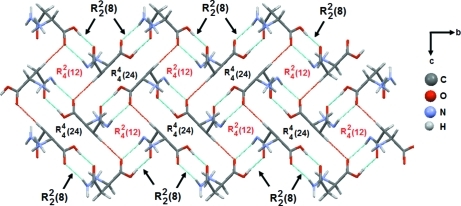
Hydrogen bonding cation-cation infinit chains within the DL-asparaginium layer. The axis a is directed downwards from the projection plane.

**Fig. 5.**
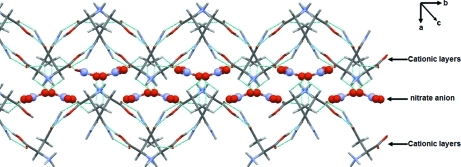
Connection between the cationic layers via cation-anion H-bonds

## supplementary crystallographic information

### Comment

DL-asparagine, the racemic melange of the aparagine L and D-enantiomers, has been used in growth-media for bacteria-growth such as Brucellae (Gerhardt & Wilson, 1948), Pseudomonas fluorescens (Palleroni et al., 1973) and lambda particles (van Wagtendonk et al., 1963).

The crystal structures of the L-enantiomer compounds are reported most often, indeed, L-asparagine monohydrate, determined by X-ray or neutron diffraction, has been reported for the first time by Kartha & de Vries (1961) and then reported, i.a., by Verbist et al. (1972); Ramanadham et al. (1972); Wang et al. (1985); Weisinger-Lewin et al. (1989); Smirnova et al. (1990); Arnold et al. (2000); Flaig et al. (2002).

Recently, Yamada et al. (2007) reported the crystal structure of the anhydrous L-asparagine and Aarthy et al. (2005) reported the crystal structure of L-asparaginium nitrate.

In the present study, the single-crystal structure determination of anhydrous DL-asparaginium nitrate as a part of the work of our team is reported. The asymmetric unit is formed by the monoprotonated asparaginium cation (C_4_H_9_O_3_N_2_)^+^ and the nitrate anion (NO_3_)^-^ (Fig. 1). Observation of the build-up of the electron density in the vicinity of O1, the different C-O bond distances [1.3116 (16) and 1.2143 (15) Å ] and the pertinent O-C-O bond angle [126.2 (1)°] clearly confirm the protonation of the carboxyl group.

The crystal structure is stabilized by O-H···O, N-H···O and C-H···O, cation-cation (Fig. 2) and cation-anion (Fig. 3) hydrogen bonds. The O1-H1···O3 cation-cation hydrogen bond is the strongest one observed in the title structure at all (Tab. 1).

The O1-H1···O3 and N3-H5N···O2 cation-cation hydrogen bonds generate a succession of infinite chains composed of R_2_^2^(8) rings that propagate in a zig-zagged way along the axis b (Fig. 4). These chains are interconnected by C3-H4···O2 hydrogen bonds (Tab. 1), giving rise to two-dimensional cationic layers which are formed by a succession of alternating R_4_^2^(12) and R_4_^4^(24) rings (Fig. 4). The former ring includes also N3-H5N···O2 while the latter O1-H1···O3 intermolecular hydrogen bonds (see Tab. 1). The cation-anion hydrogen bonds interlink the cationic layers into a three-dimensional network (Fig. 5). Some of the cation-anion N—H···O hydrogen bonds are bifurcated of the type D-H···(A_1_,A_2_) (Desiraju & Steiner, 1999).

The backbone conformation of the cation asparaginium is stabilized by the intramolecular N2-H2N···O3 interaction, with the S(6) motif (Bernstein et al., 1995), between the O atom of the amide group as an acceptor and one of the H atoms of the -NH_3_ group. The pertinent angle N2-H2N···O3 is quite acute (Tab. 1). A similiar intramolecular interaction is observed in L-asparaginium picrate (Anitha et al., 2005) and L-asparaginium nitrate (Aarthy et al., 2005).

In this study the graph-set suggested by Etter et al. (1990) and the quantitative graph set descriptor G^a^_d_(n) (Bernstein et al., 1995) are used in order to describe the hydrogen bonds in the title structure (Tab. 1). The unitary graph set  is composed of ten motifs: N_1_ = C(7)C(7)S(6)C(5)DDDDDD where C(7) applies for O1—H···O3 and N3—H5N···O2; S(6) for N2—H2N···O3 and C(5) for C3—H4···O2 while the rest of the motifs D refer to the cation-anion interactions.

### Experimental

The title compound was prepared by heating of a mixture of DL-asparagine monohydrate of purity 98 % (Alfa Aesar) and nitric acid. This mixture was obtained by dissolution and agitation for 20 minutes of 0.75 g of the DL-asparagine monohydrate in 15 ml of water at 25°C followed by addition of 15 ml of 1 *M* nitric acid. Colourless needle crystals with approximate dimensions 0.30 x 0.20 x 0.10 mm were obtained by evaporation of the solution at room temperature in the course of a few weeks.

### Refinement

All the H atoms were located in the difference electron density maps. All the H atoms attached to C were treated as riding with C-H = 1.00 Å (methine) or 0.99 Å (methylene) with U_iso_H = 1.2U_eq_C. The coordinate parameters of the H atoms attached to N or O were freely refined with U_iso_H = 1.2U_eq_N and U_iso_H = 1.5U_eq_O.

### Crystal data

**Table:**

C_4_H_9_N_2_O_3_^+^·NO_3_^−^	*F*(000) = 408
*M**_r_* = 195.14	*D*_x_ = 1.679 Mg m^−^^3^
Monoclinic, *P*2_1_/*c*	Mo *K*α radiation, λ = 0.71073 Å
Hall symbol: -P 2ybc	Cell parameters from 19446 reflections
*a* = 7.923 (2) Å	θ = 2.9–30.0°
*b* = 9.608 (2) Å	µ = 0.16 mm^−^^1^
*c* = 10.613 (3) Å	*T* = 100 K
β = 107.105 (2)°	Prism, colorless
*V* = 772.2 (3) Å^3^	0.3 × 0.2 × 0.09 mm
*Z* = 4	

### Data collection

**Table:**

Oxford Diffraction Xcalibur–Sapphire2 CCD diffractometer	2236 independent reflections
Radiation source: fine-focus sealed tube	1804 reflections with *I* > 2σ(*I*)
graphite	*R*_int_ = 0.036
φ and ω scans	θ_max_ = 30.0°, θ_min_ = 2.9°
Absorption correction: gaussian (*CrysAlis RED*; Oxford Diffraction, 2008)	*h* = −11→11
*T*_min_ = 0.966, *T*_max_ = 0.991	*k* = −13→13
19446 measured reflections	*l* = −11→14

### Refinement

**Table:**

Refinement on *F*^2^	Primary atom site location: structure-invariant direct methods
Least-squares matrix: full	Secondary atom site location: difference Fourier map
*R*[*F*^2^ > 2σ(*F*^2^)] = 0.035	Hydrogen site location: difference Fourier map
*wR*(*F*^2^) = 0.087	H atoms treated by a mixture of independent and constrained refinement
*S* = 1.07	*w* = 1/[σ^2^(*F*_o_^2^) + (0.0466*P*)^2^ + 0.1163*P*] where *P* = (*F*_o_^2^ + 2*F*_c_^2^)/3
2236 reflections	(Δ/σ)_max_ < 0.001
136 parameters	Δρ_max_ = 0.45 e Å^−^^3^
0 restraints	Δρ_min_ = −0.18 e Å^−^^3^

### Special details

**Table:**

Geometry. Bond distances, angles etc. have been calculated using the rounded fractional coordinates. All su's are estimated from the variances of the (full) variance-covariance matrix. The cell esds are taken into account in the estimation of distances, angles and torsion angles
Refinement. Refinement of *F*^2^ against ALL reflections. The weighted *R*-factor *wR* and goodness of fit *S* are based on *F*^2^, conventional *R*-factors *R* are based on *F*, with *F* set to zero for negative *F*^2^. The threshold expression of *F*^2^ > σ(*F*^2^) is used only for calculating *R*-factors(gt) *etc*. and is not relevant to the choice of reflections for refinement. *R*-factors based on *F*^2^ are statistically about twice as large as those based on *F*, and *R*- factors based on ALL data will be even larger.

### Fractional atomic coordinates and isotropic or equivalent isotropic displacement parameters (Å^2^)

**Table:**

	*x*	*y*	*z*	*U*_iso_*/*U*_eq_	
O1	0.35366 (11)	1.04131 (8)	0.62255 (8)	0.0171 (2)	
H1	0.403 (2)	1.0991 (17)	0.6820 (16)	0.0256*	
O2	0.21545 (11)	0.96178 (8)	0.76602 (8)	0.0150 (2)	
O3	0.46509 (10)	0.71971 (8)	0.71175 (8)	0.0144 (2)	
N2	0.07508 (13)	0.74096 (10)	0.61109 (10)	0.0133 (3)	
H1N	0.0164 (19)	0.7751 (15)	0.6650 (14)	0.0158*	
H2N	0.1526 (19)	0.6789 (15)	0.6612 (14)	0.0158*	
H3N	0.0026 (19)	0.6923 (15)	0.5440 (14)	0.0158*	
N3	0.57418 (14)	0.66187 (11)	0.54436 (11)	0.0172 (3)	
H4N	0.5623 (19)	0.6635 (16)	0.4621 (16)	0.0207*	
H5N	0.659 (2)	0.6100 (16)	0.5979 (15)	0.0207*	
C1	0.24959 (14)	0.95636 (10)	0.66154 (11)	0.0118 (3)	
C2	0.16701 (14)	0.84965 (10)	0.55565 (10)	0.0111 (3)	
H2	0.07526	0.89888	0.48445	0.0134*	
C3	0.29888 (14)	0.78621 (11)	0.49291 (10)	0.0129 (3)	
H3	0.34226	0.85986	0.44499	0.0154*	
H4	0.23798	0.71521	0.42775	0.0154*	
C4	0.45449 (14)	0.71962 (10)	0.59194 (11)	0.0117 (3)	
O4	0.10171 (10)	0.28059 (8)	0.69646 (8)	0.0164 (2)	
O5	0.18243 (12)	0.48467 (9)	0.78126 (9)	0.0228 (3)	
O6	0.17667 (12)	0.44085 (9)	0.57939 (9)	0.0204 (3)	
N1	0.15474 (12)	0.40279 (9)	0.68709 (9)	0.0132 (3)	

### Atomic displacement parameters (Å^2^)

**Table:**

	*U*^11^	*U*^22^	*U*^33^	*U*^12^	*U*^13^	*U*^23^
O1	0.0219 (4)	0.0149 (4)	0.0156 (4)	−0.0083 (3)	0.0074 (3)	−0.0035 (3)
O2	0.0182 (4)	0.0129 (3)	0.0149 (4)	−0.0026 (3)	0.0064 (3)	−0.0031 (3)
O3	0.0147 (4)	0.0153 (4)	0.0131 (4)	0.0045 (3)	0.0040 (3)	0.0021 (3)
N2	0.0129 (4)	0.0116 (4)	0.0153 (5)	−0.0020 (3)	0.0042 (4)	−0.0034 (3)
N3	0.0171 (5)	0.0193 (5)	0.0168 (5)	0.0066 (4)	0.0074 (4)	0.0029 (4)
C1	0.0105 (5)	0.0090 (4)	0.0144 (5)	0.0017 (3)	0.0012 (4)	0.0007 (4)
C2	0.0113 (5)	0.0096 (4)	0.0117 (5)	0.0008 (3)	0.0021 (4)	−0.0004 (3)
C3	0.0139 (5)	0.0125 (4)	0.0117 (5)	0.0029 (4)	0.0030 (4)	0.0000 (4)
C4	0.0119 (5)	0.0083 (4)	0.0151 (5)	−0.0003 (3)	0.0043 (4)	0.0010 (4)
O4	0.0158 (4)	0.0115 (4)	0.0222 (4)	−0.0018 (3)	0.0061 (3)	−0.0004 (3)
O5	0.0249 (5)	0.0228 (4)	0.0197 (4)	−0.0048 (3)	0.0049 (4)	−0.0115 (3)
O6	0.0266 (5)	0.0189 (4)	0.0205 (4)	0.0002 (3)	0.0142 (4)	0.0018 (3)
N1	0.0104 (4)	0.0127 (4)	0.0164 (5)	−0.0004 (3)	0.0037 (3)	−0.0027 (3)

### Geometric parameters (Å, °)

**Table:**

O1—C1	1.3107 (16)	N2—H3N	0.903 (14)
O2—C1	1.2173 (16)	N2—H2N	0.907 (15)
O3—C4	1.2494 (16)	N3—H5N	0.893 (16)
O1—H1	0.845 (16)	N3—H4N	0.850 (16)
O4—N1	1.2608 (14)	C1—C2	1.5198 (17)
O5—N1	1.2397 (15)	C2—C3	1.5218 (18)
O6—N1	1.2599 (15)	C3—C4	1.5072 (18)
N2—C2	1.4898 (17)	C2—H2	1.0000
N3—C4	1.3205 (18)	C3—H3	0.9900
N2—H1N	0.899 (15)	C3—H4	0.9900
			
C1—O1—H1	111.7 (11)	C1—C2—C3	113.09 (9)
H2N—N2—H3N	106.6 (13)	N2—C2—C3	111.74 (8)
C2—N2—H2N	111.5 (10)	N2—C2—C1	109.57 (9)
H1N—N2—H3N	111.3 (14)	C2—C3—C4	113.02 (9)
C2—N2—H1N	113.6 (9)	N3—C4—C3	116.35 (10)
C2—N2—H3N	108.9 (9)	O3—C4—N3	123.27 (11)
H1N—N2—H2N	104.7 (13)	O3—C4—C3	120.38 (10)
C4—N3—H4N	120.7 (11)	N2—C2—H2	107.00
H4N—N3—H5N	119.9 (15)	C1—C2—H2	107.00
C4—N3—H5N	118.8 (10)	C3—C2—H2	107.00
O4—N1—O6	118.68 (9)	C2—C3—H4	109.00
O5—N1—O6	120.55 (9)	H3—C3—H4	108.00
O4—N1—O5	120.76 (9)	C4—C3—H3	109.00
O1—C1—C2	111.14 (9)	C4—C3—H4	109.00
O1—C1—O2	126.15 (10)	C2—C3—H3	109.00
O2—C1—C2	122.67 (10)		
			
O1—C1—C2—N2	170.37 (9)	N2—C2—C3—C4	−67.79 (11)
O1—C1—C2—C3	45.00 (12)	C1—C2—C3—C4	56.40 (11)
O2—C1—C2—N2	−11.93 (15)	C2—C3—C4—O3	0.35 (14)
O2—C1—C2—C3	−137.30 (11)	C2—C3—C4—N3	179.75 (10)

### Hydrogen-bond geometry (Å, °)

**Table:**

*D*—H···*A*	*D*—H	H···*A*	*D*···*A*	*D*—H···*A*
O1—H1···O3^i^	0.845 (16)	1.736 (16)	2.571 (2)	169.0 (17)
N2—H1N···O4^ii^	0.899 (15)	1.962 (15)	2.822 (2)	159.6 (13)
N2—H2N···O3	0.907 (15)	2.406 (16)	2.965 (2)	119.9 (12)
N2—H2N···O5	0.907 (15)	2.233 (14)	3.024 (2)	145.4 (13)
N2—H2N···O6	0.907 (15)	2.474 (15)	3.039 (2)	120.7 (11)
N2—H3N···O4^iii^	0.903 (14)	2.454 (14)	3.157 (2)	135.0 (12)
N2—H3N···O6^iii^	0.903 (14)	2.068 (15)	2.957 (2)	168.3 (14)
N3—H5N···O2^iv^	0.893 (16)	2.064 (15)	2.924 (2)	161.4 (15)
C3—H3···O5^v^	0.9900	2.3600	3.086 (2)	130.00
C3—H4···O2^v^	0.9900	2.3900	3.313 (2)	156.00

Symmetry codes: (i) −*x*+1, *y*+1/2, −*z*+3/2; (ii) −*x*, *y*+1/2, −*z*+3/2; (iii) −*x*, −*y*+1, −*z*+1; (iv) −*x*+1, *y*−1/2, −*z*+3/2; (v) *x*, −*y*+3/2, *z*−1/2.
